# A GIP Receptor Agonist Exhibits β-Cell Anti-Apoptotic Actions in Rat Models of Diabetes Resulting in Improved β-Cell Function and Glycemic Control

**DOI:** 10.1371/journal.pone.0009590

**Published:** 2010-03-09

**Authors:** Scott B. Widenmaier, Su-Jin Kim, Gary K. Yang, Thomas De Los Reyes, Cuilan Nian, Ali Asadi, Yutaka Seino, Timothy J. Kieffer, Yin Nam Kwok, Christopher H. S. McIntosh

**Affiliations:** 1 Department of Cellular and Physiological Sciences and the Diabetes Research Group, Life Sciences Institute, University of British Columbia, Vancouver, British Columbia, Canada; 2 Department of Diabetes and Clinical Nutrition, Kansai Electric Power Hospital, Osaka, Japan; University of Bremen, Germany

## Abstract

**Aims:**

The gastrointestinal hormone GIP promotes pancreatic islet function and exerts pro-survival actions on cultured β-cells. However, GIP also promotes lipogenesis, thus potentially restricting its therapeutic use. The current studies evaluated the effects of a truncated GIP analog, D-Ala^2^-GIP_1–30_ (D-GIP_1–30_), on glucose homeostasis and β-cell mass in rat models of diabetes.

**Materials and Methods:**

The insulinotropic and pro-survival potency of D-GIP_1–30_ was evaluated in perfused pancreas preparations and cultured INS-1 β-cells, respectively, and receptor selectivity evaluated using wild type and GIP receptor knockout mice. Effects of D-GIP_1–30_ on β-cell function and glucose homeostasis, *in vivo*, were determined using Lean Zucker rats, obese Vancouver diabetic fatty rats, streptozotocin treated rats, and obese Zucker diabetic fatty rats, with effects on β-cell mass determined in histological studies of pancreatic tissue. Lipogenic effects of D-GIP_1–30_ were evaluated on cultured 3T3-L1 adipocytes.

**Results:**

Acutely, D-GIP_1–30_ improved glucose tolerance and insulin secretion. Chronic treatment with D-GIP_1–30_ reduced levels of islet pro-apoptotic proteins in Vancouver diabetic fatty rats and preserved β-cell mass in streptozotocin treated rats and Zucker diabetic fatty rats, resulting in improved insulin responses and glycemic control in each animal model, with no change in body weight. In *in vitro* studies, D-GIP_1–30_ exhibited equivalent potency to GIP_1–42_ on β-cell function and survival, but greatly reduced action on lipoprotein lipase activity in 3T3-L1 adipocytes.

**Conclusions:**

These findings demonstrate that truncated forms of GIP exhibit potent anti-diabetic actions, without pro-obesity effects, and that the C-terminus contributes to the lipogenic actions of GIP.

## Introduction

Glucose homeostasis is maintained in the majority of people with insulin resistance through adaptive responses in the function and mass of their pancreatic β-cells [1]. However some individuals lack the underlying genetic program to adequately adapt [2], in which case insulin responses to circulating glucose progressively deteriorate, resulting in the development of type 2 diabetes. Clinical studies have shown that β-cell function is reduced ∼50% in patients with ‘pre-diabetes’ and ∼80% in type 2 diabetes [3], and autopsy studies revealed a progressive loss in β-cell mass during disease development, with increased β-cell apoptosis being the major contributor [4], [5]. Consequently, it has recently been argued that therapeutics targeted at improving β-cell function should be implemented early in disease progression in order to increase the probability of achieving glycemic control and reducing associated morbidities [6].

Decreased β-cell function and mass in type 2 diabetes involves the generation of β-cell stress [7], [8] resulting from chronic exposure to elevated glucose and free fatty acids [9], pro-inflammatory cytokines [10] and human islet amyloid polypeptide [11]. Therapeutics counteracting these β-cell stressors should therefore have beneficial effects in patients with type 2 diabetes. The incretin hormones glucagon-like peptide 1 (GLP-1) and glucose-dependent insulinotropic polypeptide (GIP) are gut derived peptides that act on G protein coupled receptors in multiple organs [12], [13]. The best established physiological role of incretins is to potentiate meal-induced insulin secretion and incretin-based therapeutics have recently been introduced, in the form of incretin mimetics [14], [15] and inhibitors of the incretin-degrading enzyme dipeptidyl peptidase IV (DPP-IV) [16]. Additionally, since activation of receptors for GLP-1 and GIP exerts pro-survival effects on β-cells [17], incretins may also be capable of maintaining β-cell mass in diabetes.

Both GLP-1 receptor agonists and DPP-IV inhibitors improve β-cell function and glycemic control in patients with type 2 diabetes [18], but there is controversy regarding the anti-diabetic potential for GIP receptor (GIPR) agonists [12]. The main reasons for this are that many patients with diabetes exhibit greatly reduced insulin responses to GIP and that elimination of GIP signaling promotes resistance to obesity in rodents [19], [20], [21], [22], suggesting that GIPR agonists would be ineffective in restoring β-cell function and may increase obesity in patients with type 2 diabetes. However, pharmacological doses of DPP-IV resistant GIP analogs are insulinotropic in rodents that are unresponsiveness to physiological levels of GIP [23], [24]. Moreover, normalization of glycemia improves β-cell sensitivity to GIP in diabetic rats [25] and in patients with type 2 diabetes [26], [27]. Since GIPR signaling promotes survival of cultured β-cells [28], [29], [30], [31] we examined the effects of chronic treatment of diabetic rats with a long-acting DPP-IV resistant GIP analog and observed superior β-cell function and increased mass, as well as improved glycemic control. Surprisingly, although the GIP analog had comparable potency to native human GIP (GIP_1–42_) on β-cells it exhibited weak potency on adipocytes. Therefore, GIPR agonists may benefit patients with type 2 diabetes without risk of promoting obesity.

## Results

### A DPP-IV Resistant GIP Analog (D-GIP_1–30_) Demonstrates Equivalent Islet Actions to GIP_1–42_

The effects of GIP_1–42_ are transient, due to rapid N-terminal cleavage by DPP-IV [12]. However, substitution of a D-alanine (Ala) at position 2 renders GIP_1–42_ DPP-IV resistant [32], while retaining full biological activity [24]. A truncated form, D-Ala^2^-GIP_1–30_ (D-GIP_1–30_), was utilized in the current studies since GIP_1–30_ was shown to exhibit full insulinotropic activity in studies on cell lines [33].

**Figure 1:**
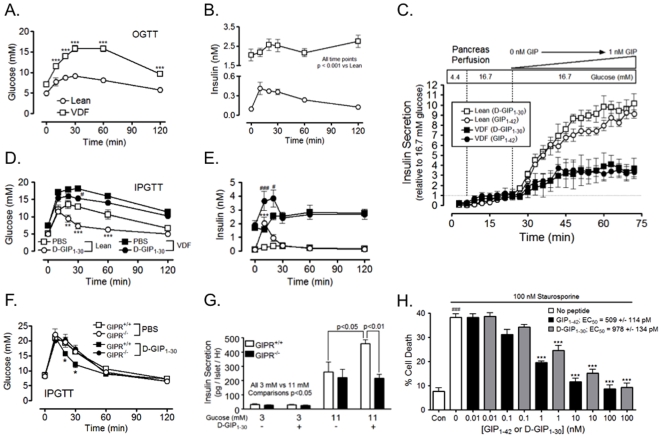
A DPP-IV resistant GIP analog (D-GIP_1–30_) demonstrates equivalent islet actions to GIP_1–42_. *A*, OGTTs were performed on fasted Lean (n = 3) and obese VDF (n = 6) rats and blood glucose levels measured. Mean ± SEM; ***, p<0.001 vs Lean rats. *B*, Insulin levels were determined from blood samples collected in *A*. Mean ± SEM with significance as indicated. *C*, Pancreas perfusions with 16.7 mM glucose + D-GIP_1–30_ or GIP_1–42_ (0–1 nM) were performed on Lean and obese VDF rats and insulin levels determined in perfusate. Mean ± SEM (n = 3). *D*, i.p. glucose tolerance tests (IPGTT) were performed on fasted Lean (n = 3) and obese VDF (n = 4) rats that received the glucose immediately following s.c. injections with PBS or D-GIP_1–30_ (8 nmol/kg BW) and blood glucose levels measured. Mean ± SEM; **, p<0.01, ***, p<0.001 vs Lean controls; #, p<0.05 vs VDF controls. *E*, Insulin levels were determined from blood samples collected in *D*. Mean ± SEM; ***, p<0.001 vs Lean controls; #, p<0.05, ###, p<0.001 vs VDF controls. *F*, IPGTTs were performed on fasted wild type (GIPR^+/+^) mice and GIPR knockout (GIPR^−/−^) mice that received the glucose immediately following s.c. injections with PBS or D-GIP_1–30_ (8 nmol/kg BW), and blood glucose levels measured. Mean ± SEM (n = 3); *, p<0.05 vs GIPR^+/+^ mice treated with PBS. *G*, Islets from GIPR^+/+^ and GIPR^−/−^ mice were incubated for 2 h in 3 or 11 mM glucose ± 10 nM D-GIP_1–30_ and secreted insulin levels determined. Mean ± SEM (n = 3); significance as indicated. *H*, INS-1 cells were treated without or with 100 nM staurosporine + increasing concentrations of D-GIP_1–30_ or GIP_1–42_ (0–100 nM) for 6 h and cell death determined. Mean ± SEM (n = 4); ###, p<0.001 vs control (no staurosporine); ***, p<0.001 vs staurosporine alone. In the upper right is the calculated EC_50_ value for GIP_1–42_ and D-GIP_1–30_.

Acute insulinotropic effects of D-GIP_1–30_ were first evaluated in Vancouver Diabetic Fatty (VDF) rats, an obese sub-strain of the Zucker Fatty rat, but with milder hyperglycemia [34]. The obese rats exhibit mild fasting hyperglycemia, but marked hyperglycemia during an oral glucose tolerance test (OGTT; Figure 1A), with elevated fasting insulin levels and blunted insulin responses (Figure 1B). Administration of linear gradients of D-GIP_1–30_ and GIP_1–42_ (0 to 1 nM) to isolated perfused pancreases from obese VDF and Lean rats, in the presence of 16.7 mM glucose, demonstrated equivalent insulinotropic potencies for the two peptides (Figure 1C). However, responsiveness of pancreata from obese VDF rats to both peptides was greatly attenuated, consistent with an earlier study [34]. In intraperitoneal (i.p.) glucose tolerance tests (IPGTT), subcutaneous (s.c.) injection of D-GIP_1–30_(8 nmol/kg BW) resulted in a moderately improved glucose profile in obese VDF rats, with profound reductions in glucose excursions in Lean rats (Figure 1D) and increased insulin responses in both obese VDF and Lean rats (Figure 1E). The specificity of D-GIP_1–30_ induced effects was assessed in GIPR knockout (GIPR^−/−^) mice. The s.c. injections of PBS or D-GIP_1–30_ (8 nmol/kg BW) immediately prior to an IPGTT (2 g glucose/kg BW) improved glucose tolerance in WT mice but not in GIPR^−/−^ mice (Figure 1F). Similarly, treatment of static mouse islet cultures with D-GIP_1–30_ potentiated insulin release from WT islets but not GIPR^−/−^ islets, and this occurred at 11 mM, but not 3 mM, glucose (Figure 1G), consistent with the glucose threshold required for GIP stimulated insulin secretion [12]. The *in vitro* effects of D-GIP_1–30_ and GIP_1–42_ on β-cell survival were also compared by monitoring the onset of cell death in staurosporine treated INS-1 cells co-treated with D-GIP_1–30_ or GIP_1–42_ (0–100 nM). Both suppressed INS-1 cell death with similar maximal effects. Although D-GIP_1–30_ demonstrated slightly reduced mean efficacy (EC_50_ values: D-GIP_1–30_ 978±134 pM vs GIP_1–42_ 509±114 pM; Figure 1H), the difference was not significant. Together these data show that D-GIP_1–30_ and GIP_1–42_ demonstrate almost identical effects on β-cells.

### Effects of D-GIP_1–30_ in Streptozotocin Treated Rats

**Figure 2:**
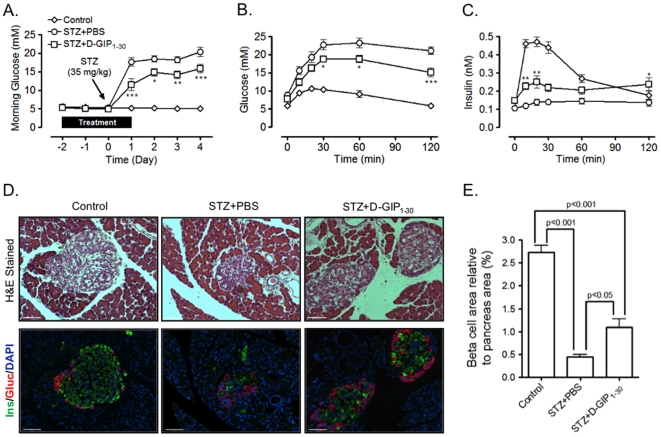
D-GIP_1–30_ partially protects β-cells in streptozotocin (STZ) treated rats. *A*, Glucose levels were monitored 2 days prior to (day −2) and 4 days (day 4) following an i.p. injection of STZ (35 mg/kg BW; on day 0) to Lean rats treated twice daily with PBS or D-GIP_1–30_ (8 nmol/kg BW) from day −2 to day 1 as well as in untreated Lean rats. *B*, On day 5, OGTTs were performed on rats described in *A* and blood glucose levels measured. *C*, Insulin levels were determined from blood samples collected in *B*. For *A–C*, Mean ± SEM (n = 4); *, p<0.05, **, p<0.01, ***, p<0.001 vs rats treated with STZ and PBS. *D*, Representative images of pancreases collected on day 6 stained with hematoxylin & eosin or with insulin (green), glucagon (red) and DAPI (blue); scale bar = 100 µm. *E*, Mean ± SEM of -cell (insulin positive) area relative to pancreas area (n = 4; 4 sections per animal); significance as shown.

The capacity for chronic GIPR activation to promote β-cell survival was then examined by determining the effects of D-GIP_1–30_ on rats exposed to the β-cell toxin, streptozotocin (STZ). Lean rats were treated twice daily with PBS or D-GIP_1–30_ (8 nmol/kg BW) from day −2 to day 1, as outlined in Figure 2A. On day 0, animals received a single i.p. injection of STZ (35 mg/kg BW) and blood glucose levels were monitored from day −2 to day 4; controls did not receive any treatment. OGTTs were performed on day 5 and pancreas samples collected for histological analysis on day 6. As expected, rats receiving STZ had elevated morning blood glucose levels and reduced glucose tolerance and insulin responses during OGTTs compared to untreated rats (Figure 2A–C). However all parameters were significantly improved in STZ treated rats receiving D-GIP_1–30_ injections, when compared to rats receiving PBS, indicating that D-GIP_1–30_ partially protected β-cells from STZ exposure. Histological analysis of pancreas samples revealed that islets in STZ treated rats had obvious structural derangements as well as apparent alpha cell expansion and increased localization to the islet core (Figure 2D). However, these derangements were much less severe in rats treated with D-GIP_1–30_. Consistent with a pro-survival effect, β-cell areas in STZ treated rats receiving D-GIP_1–30_ injections were significantly greater than those receiving PBS injections (Figure 2E and S1).

### Effects of D-GIP_1–30_ in VDF Rats

**Figure 3:**
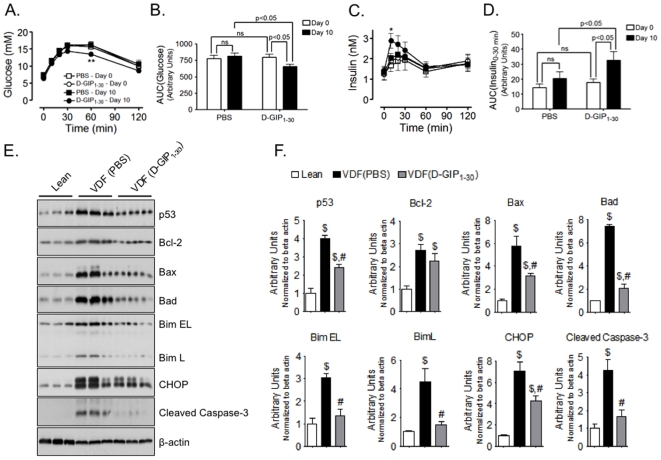
D-GIP_1–30_ improves islet function and diminishes islet pro-apoptotic protein levels in VDF rats. *A*, OGTTs were performed on obese VDF rats ∼24 h prior to and ∼48 h following 10 days of twice daily treatment with PBS or D-GIP_1–30_ (8 nmol/kg BW) and blood glucose levels measured. Mean ± SEM (n = 6); **, p<0.01 vs same VDF rats prior to treatment. *B*, Integrated glucose profile for OGTTs described in *A*. Mean ± SEM (n = 6); significant differences as shown. *C*, Insulin levels were determined from blood samples collected in *A*. Mean ± SEM (n = 6); *, p<0.05 vs same VDF rats prior to treatment. *B*, Integrated acute insulin response (from 0 to 30 minutes) for insulin profiles described in *C*. Mean ± SEM (n = 6); significant differences as shown. *E*, Islets were isolated from VDF rats and age matched Lean rats ∼24 h following OGTTs and Western analysis performed on cell lysates with indicated antibodies. *F*, For quantification, protein levels were normalized to beta-actin and expressed relative to Lean controls. Mean ± SEM (n = 3); $, p<0.05 vs Lean; #, p<0.05 vs VDF controls.

In order to establish whether chronic stimulation with D-GIP_1–30_ could evoke improvements in β-cell function, obese VDF rats were treated with s.c. injections of D-GIP_1–30_ (8 nmol/kg BW) or vehicle control (PBS) twice daily for 10 days. Although GIP is considered a ‘pro-obesity hormone’, there were no significant differences in final body weights at the end of the 10 day treatment period (Figure S2), in fact weight gain was significantly less in D-GIP_1–30_ treated (2.2±0.3 g/day) versus PBS treated (3.6±0.3 g/day) obese VDF rats. Approximately 48 h following final treatment, thus allowing complete peptide clearance from blood, OGTTs were performed, which showed that D-GIP_1–30_ treatment of obese VDF rats significantly improved glucose tolerance and acute insulin responses (0 to 30 min), whereas PBS treatment had no effect (Figure 3A–D). Following the OGTTs (∼24 h) islets were isolated and protein samples collected from PBS and D-GIP_1–30_ treated obese VDF rats along with age matched Lean rats. Western blot analysis revealed that islets from PBS treated obese VDF rats expressed significantly increased levels of pro-apoptotic proteins, when compared to Lean rats (p53, bax, bad, bim, chop, cleaved caspase-3) The anti-apoptotic protein bcl-2 was also elevated in PBS treated obese VDF rats, but only the increases in pro-apoptotic protein levels were reduced by D-GIP_1–30_ treatment (Figures 3E & F), resulting in a decrease in the bax/bcl-2 ratio.

### Effects of D-GIP_1–30_ in Zucker Diabetic Fatty (ZDF) Rats

**Figure 4:**
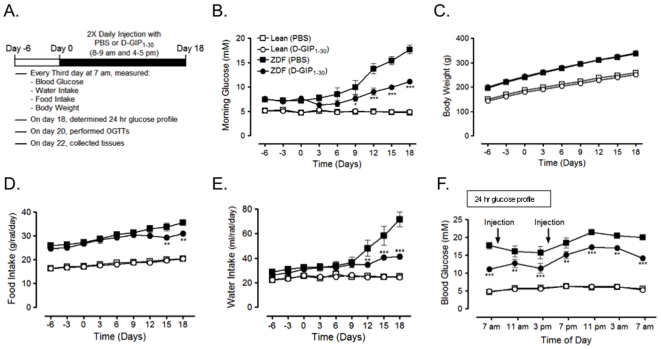
D-GIP_1–30_ improves glycemic control in ZDF rats. *A*, Schematic depicting the treatment protocol in which Lean or obese ZDF rats (starting at 6 weeks of age) were monitored every 3 days from day −6 to day 18 and treated twice daily with PBS or D-GIP_1–30_ (8 nmol/kg BW) from day 0 to day 18. *B–E*, Routine monitoring involved measurements of blood glucose (*B*), body weight (*C*), food intake (*D*), and water intake (*E*). Mean ± SEM (n = 6); *, p<0.05, **, p<0.01, ***, p<0.001 vs ZDF rats treated with PBS. *F*, On day 18, blood glucose levels were determined every 3 h over a 24 h period. Mean ± SEM (n = 6); **, p<0.01, ***, p<0.001 vs ZDF rats treated with PBS.

Since GIPR activation with D-GIP_1–30_ improved β-cell responses to glucose and β-cell survival, the effects of longer D-GIP_1–30_ treatment on glycemic control and β-cell mass were examined in male obese Zucker diabetic fatty (ZDF) rats. This model was chosen because male obese ZDF rats incur an aggressive onset of β-cell apoptosis and are one of the most commonly used and well characterized models of type 2 diabetes [35], [36]. Male Lean and obese ZDF rats (starting at 6 weeks of age) were monitored from day −6 to day 18 (see Figure 4A). Treatment with PBS or D-GIP_1–30_ (8 nmol/kg BW) began at day 0. Lean rats treated with PBS or D-GIP_1–30_ showed no significant changes in any parameter throughout the study. No differences between the groups of obese ZDF rats were observed between day −6 to day 0 but, following onset of treatment, glycemia was lower in obese ZDF rats treated with D-GIP_1–30_, reaching significance by day 9 (Figure 4B). The difference in glycemia between D-GIP_1–30_ and PBS treated groups increased over the subsequent 9 days (day 18 glucose values: D-GIP_1–30_ 11.1±0.3 mM vs PBS 17.7±0.9 mM). Body weights did not differ between obese ZDF groups (Figure 4C), but food intake was significantly reduced in D-GIP_1–30_ treated obese ZDF rats by day 15 (Figure 4D). More striking was the markedly reduced water intake in the D-GIP_1–30_ treated obese ZDF rats as early as day 12 (Figure 4E). These changes correlated with glucose levels, indicating a reduction in the onset of diabetes-induced polydipsia; polyuria was also evident in rats having polydipsia. On the final treatment day (day 18), glucose levels were monitored every 3 h over a 24 h period. Obese ZDF rats treated with D-GIP_1–30_ had significantly lower glucose levels than PBS treated obese ZDF rats at all time points (Figure 4F). Collectively this indicates that D-GIP_1–30_ exerted potent anti-diabetic effects on obese ZDF rats.

**Figure 5:**
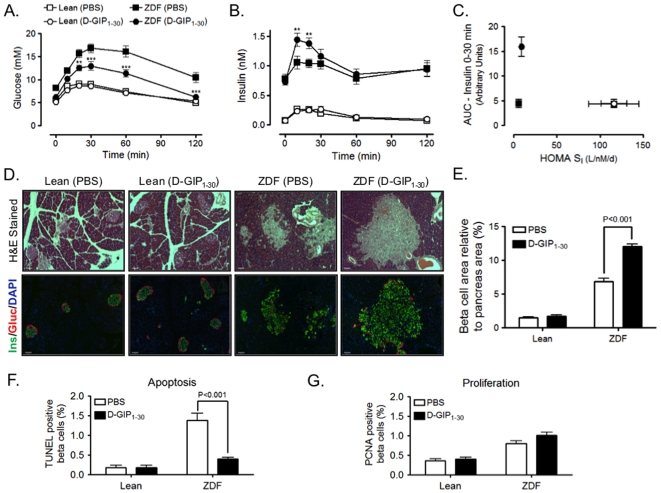
D-GIP_1–30_ improves β-cell function and mass and glucose tolerance in ZDF rats. *A*, OGTTs were performed on fasted Lean and ZDF rats (described in figure 4) ∼48 h following the last day of treatment and blood glucose levels were measured. Mean ± SEM (n = 6); **, p<0.01, ***, p<0.001 vs ZDF rats treated with PBS. *B*, Insulin levels were determined from blood samples collected in *A*. Mean ± SEM (n = 6); **, p<0.01 vs ZDF rats treated with PBS. *C*, Integrated acute insulin response (from 0 to 30 minutes) for profiles described in *B* was plotted with respect to HOMA S_I_. Mean ± SEM (n = 6). *D*, Representative images of pancreases collected ∼24 h following OGTTs. Pancreases were stained with hematoxylin & eosin or with insulin (green), glucagon (red) and DAPI (blue); scale bar = 100 µm. *E*, Mean ± SEM of β-cell (insulin positive) area relative to pancreas area (n = 3; 4 sections per animal); significance as shown. *E*, Mean percent ± SEM of β-cells undergoing apoptosis as determined via TUNEL positive nuclei (n = 6); significance as shown. *F*, Mean percent ± SEM of -cells undergoing proliferation as determined via PCNA positive nuclei (n = 6).

The anti-diabetic effects of D-GIP_1–30_ were likely a result of improved β-cell function and mass. This was examined by performing OGTTs on Lean and obese ZDF rats approximately 48 h following final injections to allow complete peptide clearance. Lean rats treated with PBS or D-GIP_1–30_ had similar glucose and insulin profiles. However, obese ZDF rats treated with D-GIP_1–30_ had reduced fasting glycemia and greatly improved glucose tolerance compared to PBS treated obese ZDF rats (Figure 5A). Importantly, D-GIP_1–30_ was so effective in obese ZDF rats that fasting and 2 h post-prandial glucose levels were similar to Lean rats. Insulin measurements revealed that obese ZDF rats had markedly elevated insulin levels compared to Lean rats, consistent with an insulin resistant phenotype (Figure 5B). However, obese ZDF rats treated with D-GIP_1–30_ had significantly greater insulin responses following glucose challenge and HOMA S_I_ calculations revealed that β-cell compensation was much greater in obese ZDF rats treated with D-GIP_1–30_ (Figure 5C). Histological analysis of pancreas samples was performed on samples collected ∼24 h following OGTTs. As expected [36], many islets from PBS treated obese ZDF rats were greatly enlarged compared to Lean rats, but with a discontinuous appearance and some alpha cell infiltration into the islet core (Figure 5D). In contrast, although most islets from D-GIP_1–30_ treated obese ZDF rats exhibited even greater enlargement (many exceeding a millimeter in diameter), they maintained structural integrity, with alpha cells residing in the islet periphery. Furthermore, β-cell area in obese ZDF rats treated with D-GIP_1–30_ was significantly greater than in those treated with PBS (Figure 5E and S3). The β-cell areas in PBS and D-GIP_1–30_ treated Lean rats were similar. Staining for apoptotic (Figure 5F) and proliferating (Figure 5G) β-cells revealed that enhanced β-cell area in D-GIP_1–30_ treated obese ZDF rats was mainly due to a significant reduction in β-cell apoptosis, although there was a modest increase in mean β-cell proliferation. Collectively these findings indicate that D-GIP_1–30_ exerted potent anti-diabetic effects in obese ZDF rats via improvements in β-cell function and mass.

### Cultured Adipocytes Differentially Respond to D-GIP_1–30_ and GIP_1–42_

**Figure 6:**
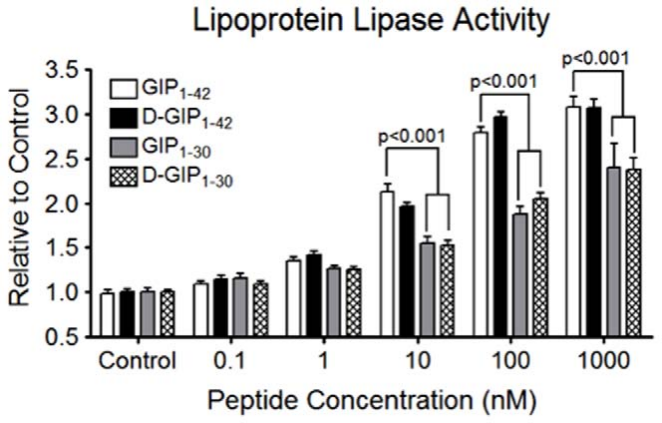
Cultured 3T3-L1 adipocytes differentially respond to D-GIP_1–30_ and GIP_1–42_. 3T3-L1 adipocytes were serum starved in 3 mM glucose DMEM containing 0.1% BSA overnight and then treated for 24 h with increasing concentrations (0–1000 nM) of GIP_1–42_, D-GIP_1–42_, GIP_1–30_, or D-GIP_1–30_ in the presence of 1 nM insulin and then LPL activity determined. Mean ± SEM (n = 7); significance as shown.

GIP has been considered a pro-obesity hormone [20] as a result of its ability to promote lipogenesis [12]. However the lack of weight gain in Lean and obese ZDF rats (Figure 4C) and reduced weight gain in obese VDF rats (Figure S2) suggested that D-GIP_1–30_ might exhibit reduced lipogenic effects, compared to GIP_1–42_. It has previously been established that GIP_1–42_ increases lipoprotein lipase (LPL) activity in cultured 3T3-L1 adipocytes [37]. In the current study, cultured 3T3-L1 adipocytes were treated with D-GIP_1–30_, GIP_1–30_, GIP_1–42_, or D-GIP_1–42_ (0–1000 nM) and LPL activity determined 24 h later. Although GIP_1–42_ and D-GIP_1–42_ promoted equivalent increases in LPL activity, D-GIP_1–30_ and GIP_1–30_ had markedly reduced effects (Figure 6); indeed, concentrations as high as 1 µM of D-GIP_1–30_ or GIP_1–30_ were unable to achieve maximal responses. The C-terminus of native GIP is therefore important for stimulatory actions in adipocytes, but not β-cells.

## Discussion

The main target for anti-diabetic therapies is a sustained reduction in glycemia, in order to lower the incidence of morbidities such as retinopathy, renal dysfunction and peripheral neuropathy [38]. Prospective studies show that many insulin resistant individuals are capable of maintaining euglycemia via compensatory responses [1], but that β-cell dysfunction and reduced β-cell mass are characteristics of those that develop type 2 diabetes [4], [6]. Therapies that improve the functional capacity and mass of β-cells should therefore offer important benefits to patients.

There is increasing evidence supporting an important role for GIPR signaling in the promotion of β-cell function and survival. Profound insulinotropic effects are achieved with physiological concentrations of GIP in normal animals [12] and with pharmacological doses of DPP-IV resistant GIP analogs in diabetic rodents [15], [23], [24]. Studies on cultured INS-1 cells and primary β-cells showed that GIPR activation promotes pro-survival responses via multiple signaling modules and reduces expression and activity of pro-apoptotic bcl-2 family proteins [28], [29], [30], [31], [39], [40]. However, although there have been extensive studies on the β-cell secretory actions of long-acting forms of GIP [20], there is a paucity of information on their β-cell protective effects. The truncated analog D-GIP_1–30_ demonstrated similar effects to the intact peptide, potentiating acute insulin responses and improving glucose tolerance in both obese VDF and Lean rats (Figure 1D&E), as well as stimulating insulin secretion from the isolated perfused pancreas preparation (Figure 1C). D-GIP_1–30_ also exhibited similar effects to GIP_1–42_ on β-cell survival in staurosporine treated INS-1 cells (Figure 1H).

It is important to note that the beneficial effects of D-GIP_1–30_ on glucose homeostasis were observed in glucose tolerance tests performed at least 48 h following the last treatment, when circulating peptide would be cleared from the circulation. These sustained responses therefore result from protective effects on islet survival, and they were observed in all three of the animal models examined. In STZ-treated rats, D-GIP_1–30_ afforded partial protection of β-cells, resulting in greater glycemic control and insulin responses (Figure 2). Protective effects of exendin-4, but not D-Ala^2^-GIP_1–42_, were recently reported in studies on STZ-induced diabetes in mice [41]. Although the reasons are not clear, a more aggressive STZ-treatment regimen was utilized compared to the current study, resulting in much greater β-cell destruction. Additionally, higher peptide dosing in their study may have also resulted in GIPR down-regulation [12] and species differences could also play a role. In the current studies on both male obese VDF (Figure 3) and obese ZDF (Figures 4&5) rats, significantly improved glycemic control and compensatory insulin responses resulted from D-GIP_1–30_ treatment. The impact of D-GIP_1–30_ treatment on diabetes progression in obese ZDF rats was also evident in the delayed onset and reduced severity of polydipsia (Figure 4E), that we attributed to the improvements in glycemia. Similarly, since GIP has not been shown to exert any major effects on food intake in rodents [12], [42], [43], the small decrease observed (Figure 4D) was likely secondary to the improved glycemia, perhaps resulting from altered hypothalamic sensing of peripherally-derived signals.

Increased β-cell area was a major factor underlying D-GIP_1–30_ induced improvements in glycemia in obese ZDF rats (Figure 5E), enabling stronger compensatory insulin responses (Figure 5C). This was mainly a result of reduced levels of β-cell apoptosis (Figure 5F) since there were no significant effects on β-cell proliferation (Figure 5G). However, previous *in vitro* studies on cultured β-cell lines [28], [44], [45] and primary islets [46] have shown that activation of GIP receptor signaling in β-cells is capable of stimulating proliferation. The lack of effect of D-GIP_1–30_ in the obese ZDF rats may be due to the elevated levels of β-cell proliferation in this model (Fig. 5G and [47]). There is, however, suggestive evidence in the literature for an effect on proliferation. Long-acting GIP analogs were found to increase islet area and number in *ob/ob* mice [48], although the relative contributions of proliferative and anti-apoptotic effects were not established. As observed with previous *in vitro* studies on GIP_1–42_ [28], [29], [30], [31], [39], [40], D-GIP_1–30_ greatly decreased islet pro-apoptotic protein levels in obese VDF rats (Figure 3E&F), an important factor in reducing β-cell loss. Bcl-2 levels were also elevated in the PBS treated obese VDF rats. However, it was the only protein examined which was not decreased by D-GIP_1–30_ treatment, resulting in an overall reduction in the bax/bcl-2 (pro-apoptotic/anti-apoptotic) ratio in response to treatment. In obese VDF rats of this age, increases in β-cell bcl-2 levels may reflect responses to the stress, thus attempting to promote survival. At any one time of tissue sampling, increases in both pro- and anti-apoptotic proteins may be detected, as compensatory responses occur. Similar observations have been previously reported, for example with apoptotic β-cells in cultured and developing rat islets [49], [50] and following serum deprivation in MIN6 β-cells [51]. It is possible, that the milder diabetes that develops in obese VDF rats, when compared to obese ZDF rats, is due to a more robust anti-apoptotic response, since the latter exhibit reduced β-cell bcl-2 levels [35], although we have no direct evidence to support this suggestion.

In developing GIP-based therapies for type 2 diabetes a major caveat has been the possibility of GIP promoting obesity [20], [22]. Such an effect would be consistent with the lipogenic actions of GIP [12] and with studies demonstrating that mice lacking functional GIP responses show resistance to the development of obesity [19], [20], [52]. However, neither mice administered long acting analogs of GIP_1–42_ [42], [43] nor GIP-overexpressing transgenic mice [53] exhibit increases in body weight, food intake, adiposity or insulin resistance, questioning whether GIPR agonists would promote obesity in patients with type 2 diabetes. Additionally, there are only weak data linking over-nutrition, GIP hypersecretion and obesity in humans [12]. Nevertheless, although the findings clearly need to be substantiated by studies on primary adipocytes and *in vivo*, the unexpected difference observed in stimulatory effects of D-GIP_1–30_ (or GIP_1–30_) and GIP_1–42_ (or D-GIP_1–42_) on LPL activity in 3T3-L1 adipocytes is intriguing (Figure 6). The high affinity binding region of GIP resides in amino acids 6–30, but the N-terminus has proven critical for actions on the pancreatic islet [12] and, as shown in the current, as well as a previous [33] study, GIP_1–30_ and GIP_1–42_ exert very similar β-cell effects. However, the C-terminal 12 amino acids have been previously shown to be important for actions on some tissues, as GIP_1–30_ exhibited much lower potency than GIP_1–42_ for inhibiting gastric acid secretion from the perfused rat stomach [54]. Since, in addition to islets and adipose tissue, GIP also appears to act as a physiological regulator in bone, the gastrointestinal tract, cardiovascular system and brain [12], there may still be tissue specific differences in responses to GIP_1–42_ and GIP_1–30_ that need to be identified. Additionally, evidence has recently been presented for the production of a C-terminally truncated version of GIP in pancreatic α-cells [55]. Therefore GIP_1–30_ may play a physiological role as either an autocrine or paracrine regulator of islet cell function and, possibly, as an endocrine hormone. Since K-cell derived GIP_1–42_ is secreted mainly during a meal, whereas α-cell secretion is elevated during the inter-digestive phase, there may be differences in the temporal activity of the two peptides; whether administration of GIP_1–30_ and GIP_1–42_ analogs during fasting and feeding results in selective tissue target effects is currently unknown. Additionally, the basis for the differential cellular activity is unclear. It is possible that the two peptides induce alternative conformational changes in the GIPR residing in different tissues, possibly due to variations in the membrane environment. However, it is more likely that cell-specific splice variants of the GIPR account for the different responses [56]. This possibility could impact on the development of clinically relevant GIP analogs.

## Materials and Methods

### Animal Studies

All studies were performed in accordance with guidelines put forth by the University of British Columbia Committee on Animal Care and the Canadian Council on Animal Care. The protocols for the experiments performed were previously reviewed, and approved, by the UBC Animal Care Committee. All studies were performed on male animals that were maintained on a 12 hr light/dark cycle with free access to standard rodent chow and water. Obese (400–500 g) and Lean (200–250 g) Vancouver Diabetic Fatty (VDF) Zucker rats (13–15 weeks of age) and C57Bl/6 GIPR knockout (GIPR^−/−^) [57] or wild type littermate mice (20–25 g; 10–14 weeks of age) were bred and maintained at the University of British Columbia (UBC). Obese ZDF (strain 370; age 4–5 weeks; 140–170 g) and Lean Zucker (strain 371; age 4–5 weeks; 110–130 g) rats were from Charles River Laboratory (Canada) and maintained at UBC for at least 1 week prior to treatment. For studies on STZ-induced diabetic rats, STZ (Sigma) was dissolved in citrate buffer (pH 4.5) and administered to animals via intraperitoneal (i.p.) injection within 15 min of dissolution. Truncated human D-Ala^2^GIP_1–30_ was synthesized by GenScript (Piscataway, NJ) and dissolved in 2% acetic acid +0.4% BSA, diluted in PBS and pH adjusted to 7.2 for administration. Identical solvent was used for control animals. During treatment periods, peptide (D-GIP_1–30_) or control (PBS) was administered at 8–9 am and 4–5 pm. Morning blood glucose was determined at 7–8 am. For glucose tolerance tests, rats were fasted for 16–17 h, challenged with 1 g glucose/kg bodyweight and blood glucose levels determined at 0, 10, 20, 30, 60, and 120 min time points. Mice were fasted for only 6 h, challenged with 2 g glucose/kg bodyweight, and blood glucose levels determined at 0, 7.5, 15, 30, 60, and 120 min time points. For all animals, blood was collected from the tail vein and glucose levels measured with a glucometer and test strips (Abbott Park, IL). Insulin levels were determined from serum samples via radioimmunoassay (RIA; Millipore™, Cat# RI-13K).

### Pancreatic Perfusions

VDF rats were deprived of food for at least 12 h, anesthetized, and pancreata isolated as previously described [58]. Arterial perfusion was achieved by cannulation of the abdominal aorta at a level adjacent to the superior mesenteric artery, while venous effluent was collected via cannulation of the portal vein. Perfusate consisted of modified Krebs-Ringer bicarbonate buffer containing 3% dextran (Sigma), 0.2% BSA (Sigma) plus 3 mM or 16.7 mM glucose gassed with 95% O_2_/5% CO_2_ and was kept at 37°C with heating units. Following a 30 min equilibration period with 16.7 mM glucose, gradients of D-Ala^2^GIP_1–30_ or GIP_1–42_ (0 to 1 nM) were administered, as described in the text. Portal vein effluent was collected in 3 min intervals at 3 ml/min with a peristaltic pump and stored at −20°C. Insulin levels were determined via RIA.

### Cell Culture for INS-1 Cells and Islet Isolation

The INS-1 β-cell line (clone 832/13) was kindly provided by Dr. C.B. Newgard (Duke University Medical Centre, North Carolina). Cells were maintained in 11 mM glucose RPMI 1640 and treated with staurosporine ± GIP_1–42_ or D-GIP_1–30_ (0–100 nM) for 6 h and % cell death determined as the number of Propidium Iodide positive cells divided by the number of Hoechst positive cells, as described in [29]. Mouse islets were isolated from pancreatic digests as previously described [59]. Islets were maintained in RPMI 1640 supplemented with 5 mM glucose, 0.25% HEPES (pH 7.4), 10% FBS, 100 units/ml penicillin G-sodium, and 100 µg/ml streptomycin sulphate. Prior to determining insulin secretion, islets (25 per well) were cultured in serum starved 3 mM glucose RPMI for 4 h and then transferred to fresh serum starved media containing 3 or 11 mM glucose ± 10 nM D-GIP_1–30_ for 2 h. Insulin secreted from islets into media was determined via RIA. For Lean and obese VDF rats, islets were isolated from pancreatic digests as previously described [60], immediately lysed, and protein samples collected for Western blot analysis.

### Cell Culture of 3T3-L1 Adipocytes and LPL Assays

3T3-L1 cells were cultured onto 96-well culture plates and induced to differentiate into adipocytes as previously described [37]. LPL enzyme activity assays were performed using the manufacturers protocol (Roar Biomedical Inc.) and presented as relative activity normalized to protein concentration.

### Western Blotting

Cell lysates were subjected to 15% SDS/PAGE and electroblotted onto nitrocellulose membrane (Bio-Rad). Antibodies used to probe membranes were all from Cell Signaling Technology (Beverly, MA) as follows: anti-beta-actin (antibody 4967), anti-bad (antibody 9292), anti-bax (antibody 2772), anti-bcl-2 (antibody 2876), anti-bim (antibody 2819), anti-caspase-3 (rabbit mAb 9665; 8G10), and anti-CHOP (mouse mAb 2895; L63F7). Immunoreactive bands were visualized by enhanced chemiluminescence (Amersham Biosciences) using horseradish peroxidase-conjugated IgG secondary antibodies. For quantification of band density, films were analyzed using densitometric software (Eagle Eye; Stratagene).

### Histological Analysis

Animals were sacrificed and pancreas samples fixed overnight at 4°C in 4% paraformaldehyde. Paraffin embedding, sectioning (5 µm), and hematoxylin and eosin (H&E) staining of samples was performed by Wax-it services (Vancouver, Canada). For immunofluorescent staining, deparaffinized and rehydrated slides underwent heat induced epitope retrieval at 95°C for 10 min in citrate buffer (10 mM citrate, 0.05% Tween 20, pH 6.0) using an EZ-Retriever™ Microwave (BioGenex, USA), and then incubated overnight at 4°C with guinea pig anti-insulin (1∶1000; Millipore), mouse anti-glucagon (1∶1000; Sigma), and/or mouse anti-PCNA (1∶200; BD Biosciences). Apoptotic cell staining with TUNEL was according to manufacturers protocol (Roche). Primary antibodies were visualized following 1 h incubation at room temperature with secondary antibodies conjugated to AlexaFluor 488 or 594 (1∶500; Molecular Probes Eugene) and then mounted in VECTASHIELD Hardset™ mounting medium with DAPI (Vector Laboratories; Cat# H-1500). Images were captured using an Axiovert 200 microscope (Carl Zeiss, Toronto, Canada) and a Retiga 2000R camera (QImaging, Burnaby, Canada) in monochrome and pseudo-coloured (fluorescent images) or in RGB format (H&E images) using the OpenLab v5.2 software (ImproVision, Lexington, USA). Following staining with 3,3′-diaminobenzidine, pancreas sections were digitally scanned using a ScanScope CS digital slide scanner and analyzed using the ImageScope positive pixel count, version 9 algorithm (Aperio Technologies Inc., USA).

### Statistical Analysis

Data, expressed as mean ± SEM, were analyzed using the non-linear regression analysis program PRISM (GraphPad, San Diego, CA). The HOMA S_I_ was determined using the methods specifically developed for ZDF rats [36]. Statistical significance of differences in mean value was tested using ANOVA with bonferroni post hoc test. A *p* value of <0.05 was considered significant.

## Supporting Information

Figure S1 Representative sections of pancreases collected from untreated rats and rats treated with PBS or D-GIP_1–30_ + STZ. Insulin positive (beta-cell) area was stained via peroxidase catalyzed reaction with 3,3′-Diaminobenzidine.
(0.46 MB JPG)

Figure S2 D-GIP_1–30_ treatment reduces weight gain in VDF rats. Bodyweights of VDF rats treated as described in Figure 3 were monitored every 2 days. Absolute body weights before and after (day 0 and 10) are shown in A and relative increases in body weight from day 0 are shown in B. Mean ± SEM (n = 6); *, p<0.05 vs VDF rats treated with PBS.
(0.30 MB JPG)

Figure S3 Representative sections of pancreases collected from Lean and obese ZDF treated with PBS or D-GIP_1–30_. Insulin positive (beta-cell) area were stained via peroxidase catalyzed reaction with 3,3′-Diaminobenzidine.
(0.44 MB JPG)
