# Supplementary figures and images for: A GIP Receptor Agonist Exhibits β-Cell Anti-Apoptotic Actions in Rat Models of Diabetes Resulting in Improved β-Cell Function and Glycemic Control
Source: PLoS One. 2010 Mar 9;5(3):e9590. doi: 10.1371/journal.pone.0009590 (PMC2834736; doi:10.1371/journal.pone.0009590)

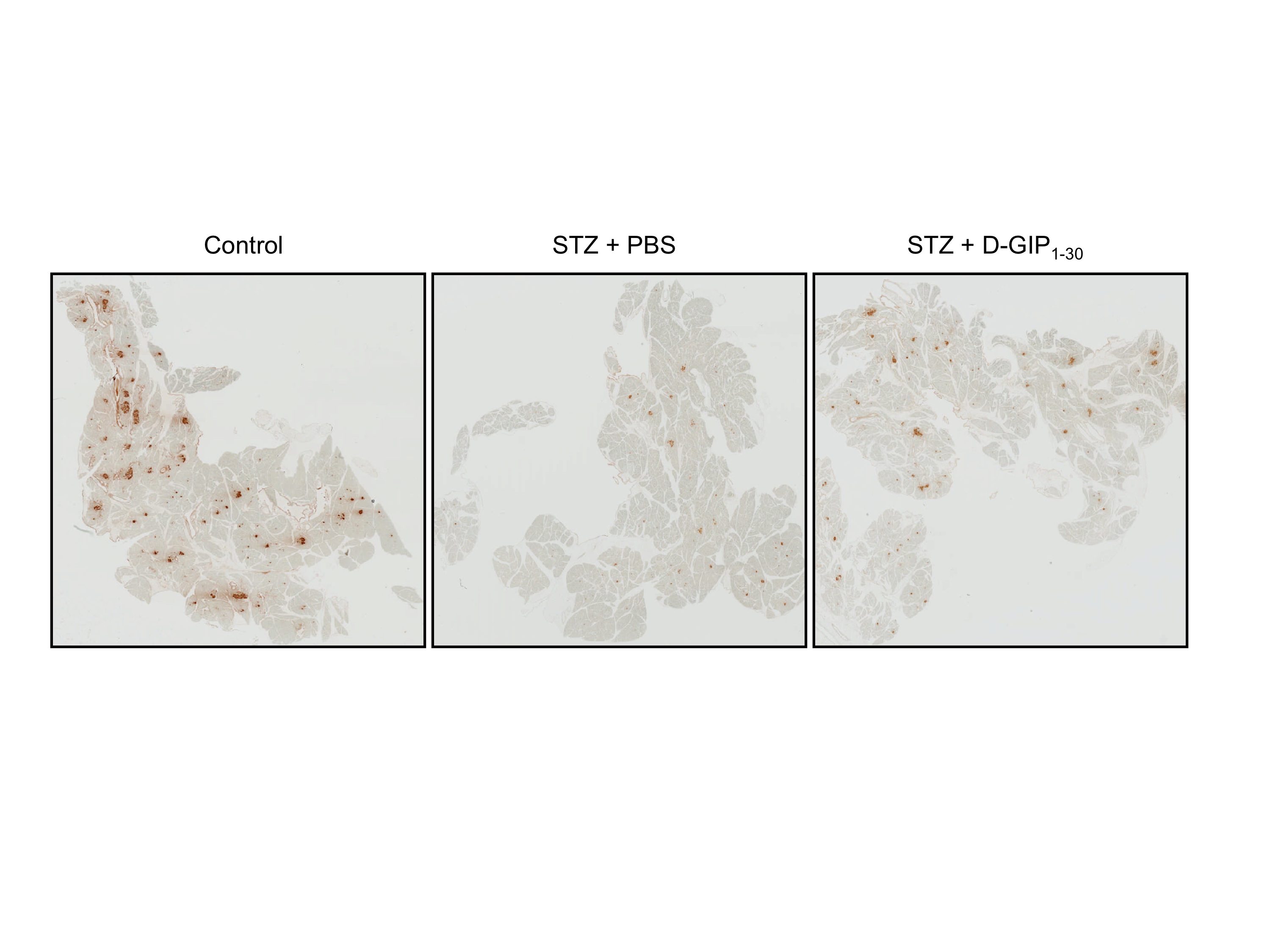

Supplement: Figure S1 — Representative sections of pancreases collected from untreated rats and rats treated with PBS or D-GIP1–30 + STZ. Insulin positive (beta-cell) area was stained via peroxidase catalyzed reaction with 3,3′-Diaminobenzidine. (0.46 MB JPG) [file pone.0009590.s001.jpg]

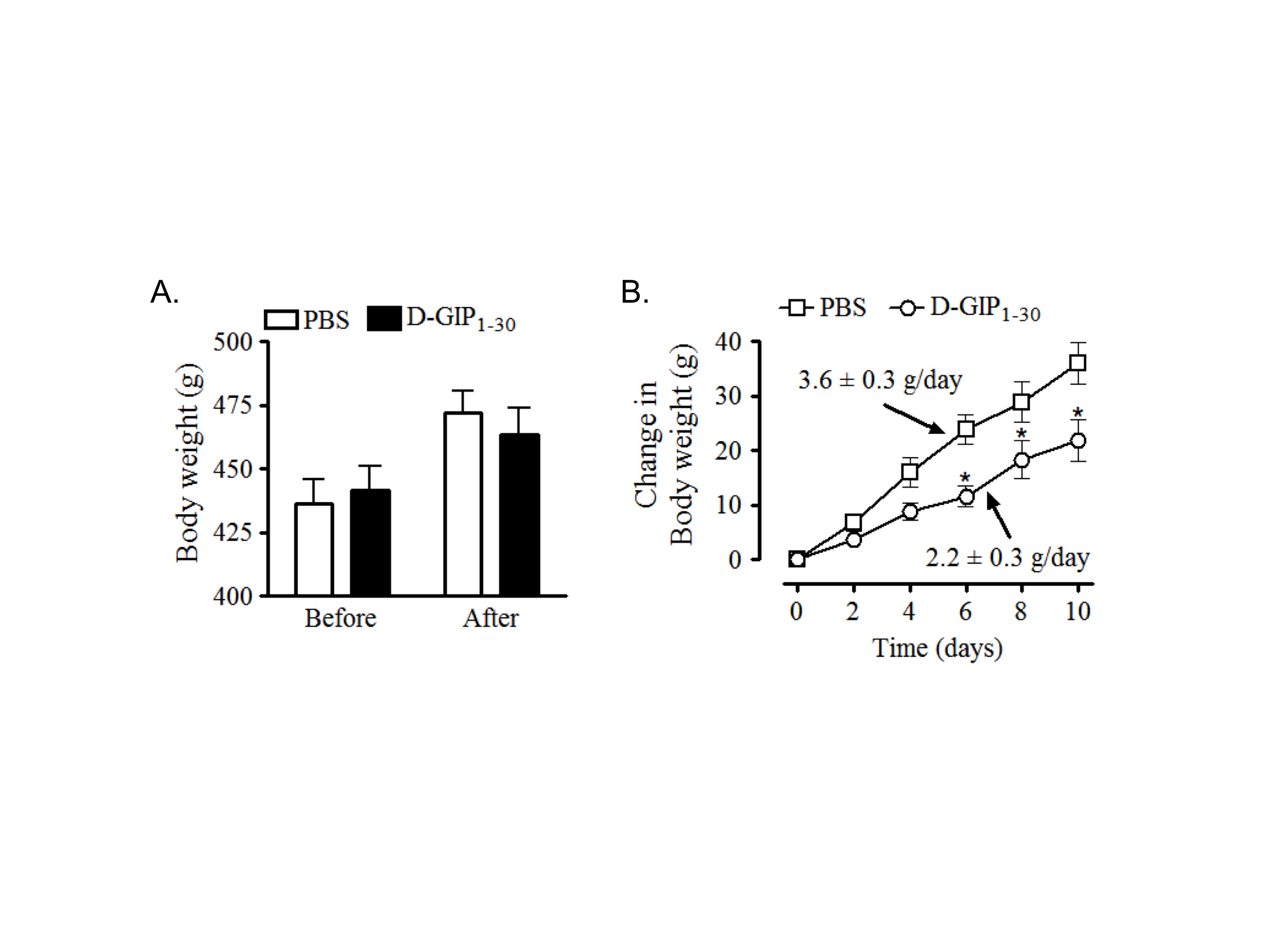

Supplement: Figure S2 — D-GIP1–30 treatment reduces weight gain in VDF rats. Bodyweights of VDF rats treated as described in Figure 3 were monitored every 2 days. Absolute body weights before and after (day 0 and 10) are shown in A and relative increases in body weight from day 0 are shown in B. Mean ± SEM (n = 6); *, p<0.05 vs VDF rats treated with PBS. (0.30 MB JPG) [file pone.0009590.s002.jpg]

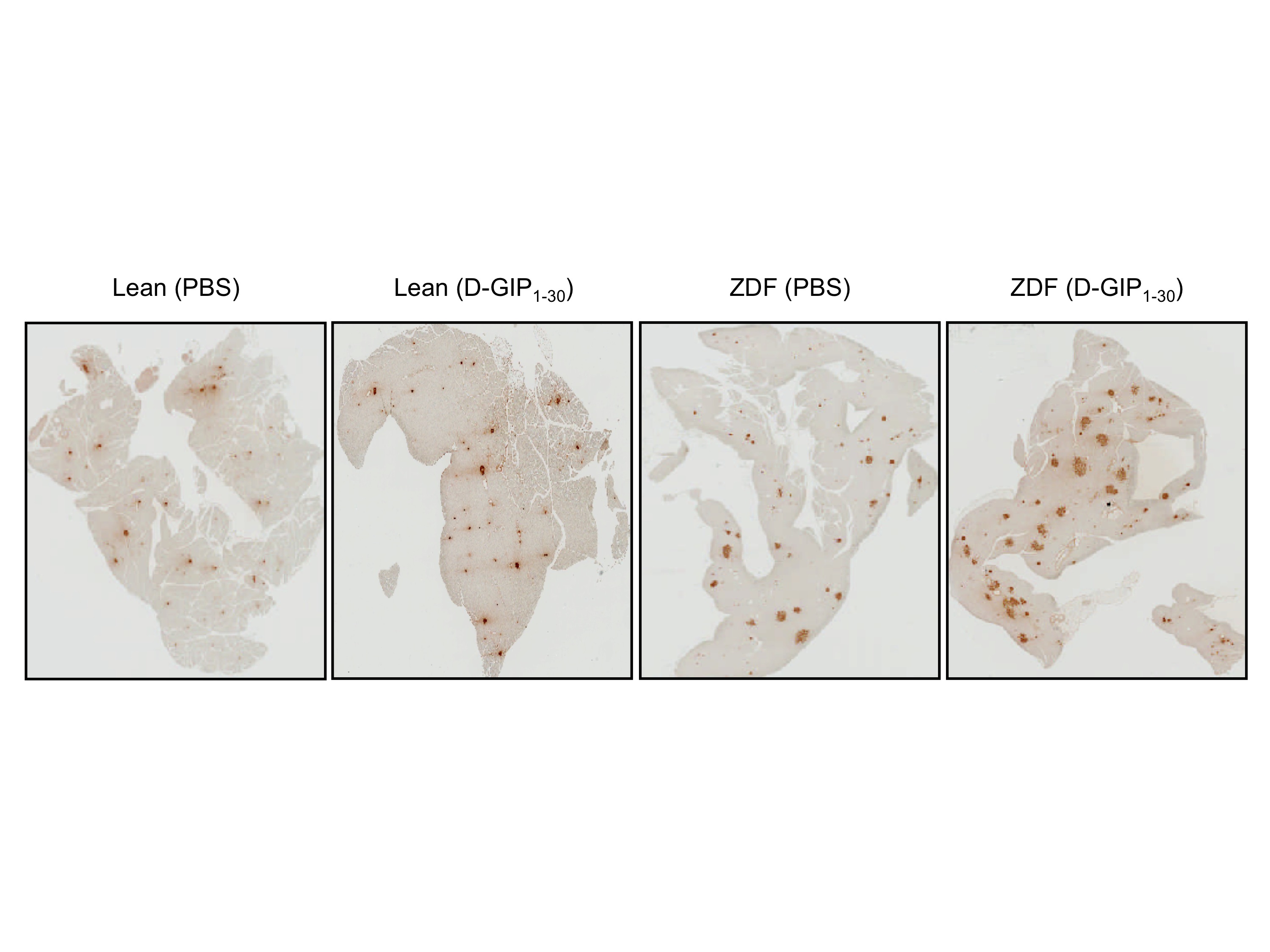

Supplement: Figure S3 — Representative sections of pancreases collected from Lean and obese ZDF treated with PBS or D-GIP1–30. Insulin positive (beta-cell) area were stained via peroxidase catalyzed reaction with 3,3′-Diaminobenzidine. (0.44 MB JPG) [file pone.0009590.s003.jpg]
